# 

**Published:** 1914-01-15

**Authors:** 


### SUBJECT INDEX TO VOLUME LXIV.

### EVENT AND COMMENT.

A Four Year Course in Dentistry, 204.
A Sensible Programme, 505.
B. J. Cigrand, 56.
Dental Brief Suspends, 1.
Dental Hygienists, 54.
Dental Dispensary Record Suspends, 103.
Dentistry for Children, 152.
Dentist Honored, 55.
Dentistry in the Polar Regions, 253.
European War, 452.
Good Hearing, 253.
Illinois Dental Society, 201.
Michigan Dental Society, 203.
National Association Bulletin, 551.
National Dental Association, Annual Meeting, 351.
National Dental Research Foundation, 552.
New Features in Dental Journalism, 54.
New Dean of California University Dental Dept. 252.
New Section of National Association, 251.
New York Medical Society, 251.
Oral Hygien's New Editor, 453.
Ohio's Contribution to the National Dental Association, 252.
Ohio State Annual Meeting, 2.
Prevention of Malocclusion, 151.
Prevention of Dental Caries, 314.
Preventive Medicine, 358.
Reorganization of N. Y. State Dental Society, 316.
Scheme to Finance Oral Hygiene Movement, 151.
Sixth International Dental Congress, 451.
Something New in Dental Legislation, 401.
Some Things to be Considered, 101.
Strike of German Dental Students, 51.
The Dental Research Problem, 313.
The Four Year Dental Course, 501.

### FORMAL PAPERS.

Anesthesia in Dentistry, D. Fyfee, L.D.S., 373.
An Appeal for Higher Art in Amalgam Fillings, Wm. R. Pond, 127.
An Important Epoch in Medical Education, 525.
A Plea for a More Intimate Knowledge of Drugs, E. B. Hosom, 57.
Are the Capillaries of Enamel Capable of Sustaining Metabolism, Theo von Beust, 180.
Buccal Protozoa, E. C. Kirk, 469.
Cause and Cure of Pyorrhea Alveolaris, H. E. Bliler, 516.
Concerning the Etiology of Dental Caries, C. F. Bodecker, 415.
Conductive Anesthesia, J. E. Nyman, 522.
Dental Exhibit at Panama-Pacific Exposition, W. H. F. N. Debille, 175.
Editor C. N. Johnson's Pledge, 475.
Extensive Occlusal Restorations, W. D. N. Moore, L.D.S., 420.
Fear and Anesthesia, Louis Schultz, 556.
Food Supply of the Child, Walter H. Jackson, 554.
Inspection of Dental Offices by the State Board of Health, 565.
Is Modern Dentistry a Specially Developed Branch of Medicine, Otto Zsigsmondy, 426.
Local Anesthesia for Major Operations on the Face and Head, Leo Eloesser, M. D., 112.
Many More Dentists Needed, Alfred P. Lee, 342.

Mixing and Packing Amalgam, W. E. Harper, 558.
New Things in Dental Pathology, O. E. Inglis, 512.
Oral Malignant Growths, Julio Endelman, 28.
Pickerill's Work on Dental Caries, Guy S. Millbury, 105.
Preventive Dentistry, Henry A. Kelley, 317.
Prevention of Dental Caries, F. Winchester, 256.
Public School Dental Clinics in Every Community, W. G. Ebersole, 159.
Pyorrhea and its Reflexes, R. L. Tower, 471.
Restoring Occlusion in Mutilated Arches, Henry W. Gillett, 177.
Retention of Upper Dentures Loomis P. Haskell, 577.
Rosin Solution in Root Filling, J. R. Callahan, 506.
Safeguarding Anesthesia, C. S. Tuttle, 338.
Shock After Operations, 170.
Sodium and Potassium, R. Ottolingui 465.
Some Facts, Chemical and Otherwise about Dentifrices, Howard C. Kelly, 328.
Some Public Aspects of the Wood Alcohol Industry, 182.
Some Recent Investigations as to Cause of Dental Caries, J. Sim Wallace, 404.
Some Thoughts Suggested by the Austro - Hungarian Problem, Dorothy Richardson, 430.
Stick to Your Teeth, 455.
Syphilis and its Relations to the Oral Cavity, H. F. Parks, 206.
The Availibility of Nutrients from Plant Sources, 166.
The Diagnosis of Malignant Diseases of the Mouth, W. J. Roe, 569.
The Early Evidences of Malocclusion and Treatment, Milton T. Watson, 154.
Teething as a Cause of Disease in Infancy, Charles Herrman, 24.
The Dosage of Cocaine and Other Drugs Used for Anesthesia, Albert H. Miller, 78.
The Family Dentist, A. E. Webster, 436.
The Function of the Dentist in Race Betterment, C. N. Johnson, 220.
The Leger-Dorez System of Adjustable Bridge Attachments, Joseph Nolin, 210.
The Necessity for Scientific Dental Research Work, A. R. Melendy, 7.
The Preservation of the Teeth, Norman Roberts, 347.
The Prophylactic Value of Gymnastics of the Jaws, Dr. Kleinsorgen, 217, 562.
The Service of Medicine to Civilization, V. C. Vaughan, M. D. 378.
Treatment of Loose Teeth Due to Inflammatory Degeneration of the Gums and Alveolar Process, Joseph Head, 64.
Treatment of Oral Infection in its Relation to Disease, 359.
The Treatment of Dental Caries in Children, F. St. J. Steadman, 265.
The Value of Formaldehyde in Septic Root Canals, F. W. Frohm, 32.
Warrented Dental Treatment, Henry Schwamm, 482.
Why So Many of Us Fail as Dentists and Business Men, W. H. Chilton, 477.

BIBLIOGRAPHY

Boardman's Biography of Members of Massachusetts Dental Society, 487.
Dalton's Essentials of Orthodontia, 486.
Dental Electro Therapeutics, 132.
Dental Jurisprudence, 487.
Dental Radiography, 36.
Dewey's Orthodontia, 579.
Dorlands Dictionary, 133.
First Aid in Dentistry, Ryan, 277.
Fischer's Local Anesthesia, 484.
Gray's Anatomy, 133.

Hughes' Questions on Prosthetic Dentistry, 579.
Operative Technics, McGhee, 131.
Practical Manual of Dental Castings, 352.
Proceedings, Institute of Dental Teachers, 277.
Prosthetic Articulation, Clapp, 485.
Research Papers, 486.
Rosin Solution for Root Filling, Callahan, 278.
Schuhmann's Question Compend, 527.
Stedman's Dictionary, 580.
Transactions National Association, 277, 351
Wilson's Dental Prosthetics, 527.

OBITUARY.

George W. Cook, 82.
Ferdinand J. T. Gorgas, 527.
John Nathan Crouse, 80.
Morgan H. Fletcher, 225.
Jacob F. Frantz, 186.
George Edwin Hunt, 439.
Samuel T. Kirk, 399.
Charles A. Meeker, 136
John W. Moffitt, 229.
Charles H. Rosenthal, 134.

INDENTS.

A Broader and Better Dental College, 536.
Accurate Morsal Surfaces in Porcelain Crowns, 398.
Adapting Porcelain Crowns with Cement, 45.
Administering Gas, 190.
Administering Calcium, 192.
Advantages of the Porcelain Inlay, 359.
A Good Syringe, 533.
A Hint Regarding the Use of the Rubber Dam, 193.
Alcoholic Drinks Favor Dental Caries, 138.
Alopechia of Dental Origin, 141.
Aluminum Solder, 44.
Alundum Polishing Powder, 394.
Alveolar Abscess with Fatal Termination, 139.
American Red Cross in European War, 447.
Analgesics on the Wane,—Vacines Coming, 40.
Anesthesia of Dentine with Albargin, 490.
Anesthesia of the Mandible, 449.
A Method of Treating Pyorrhea, 589.
A New Protective Feature, 89.
A Physiological Hemostatic, 284.
Annual Dental Registration Fee, 242.
An Anti-Oxidizer, 235.
Antiseptic Varnish for Surgeons, 447.
Arresting Hemorrhage Following Extracting, 235.
Arsenic in Temporary Teeth, 238.
Arsenic Pentoxid, 586.
Automobile a Cause of Caries, 141.
Bacteria Metabolism and Internal Medicine, 360.
Bad Teeth, 280.
Baldness from Affected Teeth, 279.
Broader Conception of Technical Operations, 138.
Broaching the Pulp, 280.
Bromural, 86.
Bulk in Porcelain Inlays, 357.
Burnishing the Gold Inlay, 359.
Care of Deciduous Teeth, 234.
Care of the Operators Hands, 356.
Casting Aluminum, 533.
Caustic Applicators, 240.
Cause of Broken Facings from Backings, 139.
Caution in Casting Aluminum, 590.
Cavity Preparation, 582.
Cement-Amalgam Filling, 355.
Classification of Oral Sepsis, 46.
Clean Impression Trays, 498.
Cleanliness in Serving Food, 241.
Cleansing and Sterilizing Solution, 355.
Clean Teeth, 445.
Condition of Blood in Hemophilia, 198.
Contact Points, 541.

Danger of Breaking Down Infected Tissue Around the Tooth, 85.
Decay of Children's Teeth, 582.
Dental Dispensary for Albany Schools, 233.
Dental Education of the People, 583.
Dental Infection, 391.
Dentists Neglectful of Aseptic Practice, 90.
Dental Mutilation by Savages, 137.
Dental Public Service, 44.
Desensitizing Sensitive Dentine, 358.
Diseased Teeth a Cause of Tuberculosis, 288.
Disinfection, 285.
Disinfection of the Hands, 198.
Doctors Caught in an Anti-spitting Campaign, 196.
Dr. Haskell as a Traveler, 46.
Early Orthodontia, 444.
Easy Method of Coating Models with Foil before Vulcanizing, 444.
Economic Sterilizer for Small Dental Instruments, 391.
Expensive Sandwich, 237.
Experimental Evidence of Variations in Alimentary Secretions, 239.
Facilitating the Taking of Impressions, 396.
Failure in Casting, 353.
Fatal Case of Sepsis, 46.
Fatalities from Salvarsan, 448.
Filling for Absorption in Pyorrhea Treatment, 588.
Filing Small Wire Rods in a Lathe, 391.
Filling Root Canals, 282.
Fixing the Fee, 42.
Germany's Experience with School Dentistry, 582.
Gift for Hospital, 391.
Gold Fillings in Artificial Teeth, 358.
Gold Shell Crowns, 353.
Gout and Pyorrhea Alveolaris, 191.
Grading its Dental Servants, 584.
Homophilia Treated by Calcium Lactate, 88.
How to Fill Root Canals, 393.
Hygienic Care of the Tooth Brush, 137.
Impressions of Irregular Teeth, 396.
Importance of a Complete Diagnosis, 495.
Importance of Mutilated Occlusion, 138.
Indications for the Amount of Oxygen Required in Nitrous Oxid and Oxygen Anesthesia, 534.
Inhibitive Anesthesia, 244.
Inlay Cement, 45.
Inlay Investment, 354.
International Congress Delegates, 583.
Iodoform Deodorizer, 583.
Laying Up Trouble, 235.
Local Anesthetic Without Cocaine, 234.
Locating Root Canals, 397.
Making Faces as an Aid to Health, 197.
Many Seals Sold in Ohio, 446.
Matching Teeth in the Mouth, 587.
Medical Endowment, 236.
Medical Schools and Graduates, 236.
Medical Education in the United States, 233.
Metal Spraying Process, 140.
Mixing Synthetic Cement, 395.
Model Cement, 499.
Mouth Disinfection with Ultra-Violet Rays, 236.
Narcosis and Lack of Oxygen, 39.
Navy Dental Surgeons, 583.
Neothesin—A New Obtunder of Sensitive Dentine, 540.
New York Dentists May Not Prescribe Cocaine, 38.
Nitric Acid as a Solvent of Vulcanite, 583.
Open the Window, 243.
Oral Sepsis a Cause of Heart Trouble. 235.
Polishing Aluminum, 538.
Polishing Vulcanite Dentures, 498.
Porcelain Inlay Matrix, 89.
Practical Eugenics, 195.

Preparing Gold Inlay Waxes for Investment, 357.
Preserving Mercury, 446.
Protecting the Pulp under Silicate Cement Fillings, 587.
Pulp Devitalization Condemned, 497.
Pulp Extirpation, 243.
Pumice in Pyorrhea Pockets, 46.
Pyorrhea Alveolaris Treatment, 286.
Pyorrhea Technic, 584.
Radium Mouth Wash for Pyorrhea, 279.
Relieving Undue Pressure by a Denture, 42.
Responsibility in Dental Practice, 445.
Right Amount of Rubber in the Flask, 86.
Safe and Easy Method of Applying Silver Nitrate to the Teeth, 587.
Saliva Tubes, 279.
Salal in Dentifrices, 89.
School Dental Dispensaries for Chicago, 199.
Securing Perfect Wax Models for Inlays, 498.
Selling Services Rather than Fillings, 280.
Setting up Full Dentures Without an Articulator, 88.
Short Broaches, 45.
Softening Inlay Wax, 45.
Soldering Backings to Platinum Pin Teeth, 194, 284.
Speed in Surgery under Anesthetics, 539.
Splicing Rubber Tubing, 281.
Statistics on Dental Caries.
Sterilization of the Mouth, 39.
Subscription Swindler at Work, 447.
Supporting Loose Teeth. 450.
Syndicalized Surgery Prophesied, 537.
Systemic Infection from the Mouth, 588.
Taking the Bite, 356.
Teeth and the Dangerous Trades, 531.
Tempering Glasses, 281.
Ten Hygienic Truths, 534.
Ten Years Progress in Medical Education, 529.
The Business Side of Dentistry. 354.
The Constitution of Metals, 393.
The Dental Nurse, the Assistant and the Mechanic, 281.
The Ductless Glands and the Teeth, 240.
The High Cost of Living a Factor in Race Degeneracy, 193.
The Importance of Frequent Examination of Wells, 194.
The Selection and Application of Flux in Soldering, 397.
The Short Road to Success, 489.
The Use of Floss Silk in the Daily Dental Toilet, 446.
Tobacco Smoking and Mental Effiency, 241.
Too Many Doctors in Austria, 242.
Too Many Patients, 280.
Tooth Detergents, 492.
Tooth Conservation Injurious, 41.
To Remove Teeth from Vulcanite Without Injury, 498.
Treatment for Hydrofluoric Acid Burns, 195.
True Recreation, 142.
Unslaked Lime for Desensitizing Dentine, 397.
War and the Drug Supply, 493.
Who Can Take an Anesthetic, 535.
Why not Stamp Out Typhoid, 197.
Value of Buccal Flanges, 28[illegible].
Value of Mask Over Su[illegible]geon's Mouth, 497.
Varnish for Plaster Casts, 354.
Vinegar for Softening Plaster, 358.

BIOGRAPHIC.

Allen, C. C., 44, 288.
Barker, D. W., 446.
Best, E. S., 357.
Black, A. D., 542, 588.
Blackmarr, C. B., 263.
Bliler, H. E., 516.
Bodecker, C. F., 415.
Brenner, Geo. P., 88.
Brown, C. E., 358.
Brubacker, Dr., 191.

Buckley, Dr., 541.
Bunting, R. W., 445.
Bush, Dr., 280.
Callahan, J. R., 506.
Cattell, D. M., 12.
Cigrand, B. J., 56.
Chilton, W. H., 477.
Cook, Geo. W., 82.
Crossland, J. H., 359.
Crouse, J. N., 80.
Crow, G. M., 537.
Cuttrell, A. J., 22.
DeBille, W. H. F. N., 175.
Dittmar, G. W., 239.
DuBois, C. C., 195.
Ebersole, W. G., 159.
Elleoser, Leo, 112.
Endelman, Julio, 28, 288.
Eustice, E., 499.
Faro, Dr., 140.
Fette, Dr. Geo. F. 586.
Fleischman, D., 286.
Fletcher, M. H., 225.
Floxas, Dr., 138.
Foreman, J. R., 264.
Frohm, F. W., 32.
Frantz, J. F., 186.
Funcke, Dr., 137.
Fyfee, Dr., 373.
Gethrow, Dr., 284.
Gillett, H. W., 138, 177.
Goldie, G. J., 398.
Goslee, Hart J., 534.
Green, A. B., 261.
Guilford, S. H., 499.
Hanauer, Dr., 532.
Hartzell, T. B., 85.
Haskell, L. P., 289.
Hayes, H. H., 89.
Head, Joseph, 64
Hermann, C., 24.
Hoff, N. S., 261.
Hofheinz, Dr., 46.
Hogan, W. J., 244.
Hoggan, J. A. C., 444.
Hosem, E. B., 264.
Howlett, F. W., 262.
Hunt, C. E., 439.
Hurtz, J. N., 193.
Inglis, O. E., 512.
Jacquet, Dr., 141.
Jenkins, N. S., 358, 360.
Johnson, C. N., 220, 240, 475.
Kelly, H. C., 328.
Kelley, H. A., 317.
Kile, C. S., 444.
Kirk, E. C., 469.
Klein, J. A., 397.
Kleinsorgen, Dr., 217.
Kloeser, Dr., 188.
Lederer, W. J., 356.
Lee, A. P., 342.
Le Gro, A. L., 399.
Lewis, J. M., 235.
Lewis, H. R., 354.
Lyons, C. J., 261.
McMillen, J. D., 45.
McGee, Dr., 497.
McKesson, E. I., 534
Melendy, A. R., 7.
Merritt, W. E., 263.
Millbury, G. S., 105.
Miller, A. H., 78.
Moffitt, J. W., 229.
Moore, W. D. N., 420.
Newell, V. B. 589.
Nies, F. H., 395.
Noel, L. G., 15.
Nolin, J., 210.
Nyman, J. E., 522.
Ottolengui, R., 445, 465.
Parks, H. F., 206, 260.
Pickerill, H. P., 239.
Pond, W. R., 127.
Puterbaugh, P. G., 354.
Richardson, Dorothy, 430.
Roberts, Norman, 347.
Sandy, B., 48.
Schultz, Dr., 540.
Schwamm, H., 482.
Smith, A. G., 396.
Smith, Dr. 588.
Smith, Dr. Julian 585.
Smith, R. E., 198.
Steadman, F. St. J., 265.
Taylor, L. C., 356.
Tower, R. L., 471.
Towner, J. D., 18.
Trueman, W. H., 41, 42, 189, 354, 355, 357.
Tuttle, C. S., 338.
Vaughan, V. C., 194, 378.
Von Beust, Theo., 180.
Walker, J. S., 281.
Wallace, J. Sim, 404.
Watson, M. T., 154.
Way, Geo. F., 192.
Webster, A. E., 282, 436.

Wellman, A. C., 45.
Wenker, R. J., 87, 90.
Wilkes, A. M., 46.
Wilkinson, F. H., 280.
Wilson, Dr. 590.
Wilson, Geo. H., 539.
Winchester, F., 256, 265.
Wood, R. J., 353.
Wooley, Dr., 393.
Wright, F. L., 396.
Zader, F. 587.
Zsigmondy, Otto, 426.

ANNOUNCEMENTS.

American Academy of Oral Prophylaxis, 544.
American Institute of Dental Teachers, 144, 591.
American Miller Memorial, 249.
Arizona Dental Society Officers, 91.
Arizona Board of Examiners, 399.
Chicago Dental Society, 591.
Dedication Forsyth Infirmary, 544.
Dinner to Dr. Weisse, 143.
Federation Dentaire Internationale, 499.
First Pan-American Dental Congress, 362.
Illinois State Society Officers, 399.
Indiana Dental Association, 92.
Indiana State Board of Dental Examiners, 545.
Kentucky Dental Association, 91.
Meeting of the National Association of Dental Faculties, 543.
[illegible]emorial Resolutions Adopted By the Indianapolis Dental Society, 549.
Michigan Board of Examiners, 247, 500.
Michigan State Society Officers, 246.
Michigan Licentiates, 248, 500.
Minnesota Dental Society, 48.
Montana Board of Examiners, 591.
National Association of Dental Faculties, 591.
National Association of Faculties, 48, 543.
National Dental Association, 249, 296, 298.
National Dental Protective Association, 200.
National Dental Relief Fund, 592.
New Jersey State Board, 200.
Northern Indian Society, 400.
Northern Ohio Dental Association, 247.
Ohio State Dental Society, 544.
Ohio State Society Officers, 500.
Panama-Pacific Congress, 49, 94, 290, 363, 545, 547.
Prize Contest, 91.
Relief Insurance, 542.
Sixth International Dental Congress, 97, 144.
S. A. Crocker & Co., Removal, 143.
South Western Michigan Dental Association, 91.
Tennessee Dental Association, 246, 295.
Texas Dental Association, 48.
The Panama Pacific Dental Congress, 545, 547.
The West Virginia State Dental Society, 545.
University of Iowa Alumni Association, 400.
Xi Psi Phi Fraternity, 295.
W. G. Ebersole.

### ANNOUNCEMENTS.

### National Association of Dental Faculties.

The annual meeting of the National Association of Dental Faculties will be held at the Iroquois Hotel, Buffalo, N. Y., January 26, 1914.

Executive Committee meeting 9:00 A. M.; general session at 10:00 A. M.

Holly B. Smith, Chairman Executive Com.
Charles Channing Allen, Sec'y.

### Minnesota State Dental Society.

The thirty-first annual meeting of the Minnesota State Dental Association will occur in Duluth, August 6-7-8, 1914, at which time the officers of the Society unite with the Duluth men in promising a most instructive and enjoyable meeting.

Benjamin Sandy, Sec'y.

### Meeting Texas State Dental Association.

The thirty-fourth annual meeting of the Texas State Dental Association will be held at Fort Worth, Texas, April 13, 14, 15, 16 and 17th, 1914.

In addition to the regular program, the Oklahoma Post-Graduate plan will be tried at this meeting.

Dr. Geo. H. Wilson of Cleveland will present Prosthesis with special reference to anatomical occlusion, and Dr. Frank H. Skinner of Chicago, Pyorrhoea, Prophylaxis and Removable Bridge-work in connection with same.

For information relative to space for exhibits, or as to clinics, address Dr. W. H. Nugent, Fort Worth, Texas. Any other information will be cheerfully furnished by the Secretary.

FRANK FORMAN, Prest.,
Waco, Texas.
J. G. FIFE, Secy.—Treas.,
Dallas, Texas

OPENS AUG. 30TH, 1915

### THE PANAMA PACIFIC DENTAL CONGRESS.

### A NEW YEAR'S ANNOUNCEMENT.

The Committee of Organization of the Panama-Pacific Dental Congress is pleased to greet the dental profession of the world with the announcement that now, twenty months before the date of opening, August 30th, 1915, the Panama-Pacific Dental Congress is so well advanced in the work of organization, that the ultimate success of the Congress is fully assured.

Executive Committees in almost every State and Country in the world are giving publicity to the Congress, securing memberships, and soliciting contributions to it's program.

The officers of the ten sections into which the program of the Congress is divided, have been selected, the list including many men of world wide prominence and recognized ability, and under their supervision a program is being prepared which will outrank in scientific value and literary excellence the program of any previous Congress.

Fifteen hundred front feet of space has already been reserved by manufacturers and dealers who will present to the Congress the greatest exhibition of dental wares and appliances, and the most comprehensive manufacturer's clinics ever assembled, and the list of exhibitors is still far from complete, and is increasing in number every week.

The Committee has distributed nearly four hundred thousand stickers bearing the Seal of the Congress and the date on which it will open, and they are becoming familiar to every user of dental supplies.

The entire Congress, Academic sessions, exhibits and clinics, will be held under one roof, in ample accommodations, in the new Auditorium now being erected in San Francisco's Civic Center, an Auditorium which, in size, architectural beauty, and peculiar fitness for the purposes it will serve, will rank with the best of similar buildings yet erected.

Letters received by the Committee from every Country in the world evince the wide-spread interest already manifested in the Congress and assure a large attendance.

The Panama-Pacific International Exposition will attract the people of the world to San Francisco in 1915; the Panama-Pacific Dental Congress will bring together at the same time the best the dental world has of men, methods and materials; to miss the Congress will be to miss the opportunity of a life time to become familiar with modern dentistry in all it's branches.

The Committee of Organization extends a hearty New Year greeting of good wishes to the dental profession, and invites you to be present in San Francisco, August 30th to September 9th, 1915.

